# Lactate Dehydrogenase B Is Required for Pancreatic Cancer Cell Immortalization Through Activation of Telomerase Activity

**DOI:** 10.3389/fonc.2022.821620

**Published:** 2022-05-20

**Authors:** Ruiguan Wang, Jiangbo Li, Changjian Zhang, Xin Guan, Boyu Qin, Rui Jin, Lingmei Qin, Shanrong Xu, Xiaona Zhang, Rong Liu, Qinong Ye, Long Cheng

**Affiliations:** ^1^ Department of Cell Engineering, Beijing Institute of Biotechnology, Beijing, China; ^2^ Faculty of Hepato-Pancreato-Biliary Surgery, Chinese People's Liberation Army (PLA) General Hospital, Beijing, China; ^3^ Department of Hepatobiliary Surgery, the Eight Medical Center, Chinese People's Liberation Army (PLA) General Hospital, Beijing, China; ^4^ Senior Department of Otolaryngology-Head & Neck Surgery, the Sixth Medical Center, Chinese People's Liberation Army (PLA) General Hospital, Beijing, China; ^5^ Strategic Support Force Medical Center, Beijing, China; ^6^ Department of Medical Oncology, the First Medical Centre, Chinese People's Liberation Army (PLA) General Hospital, Beijing, China; ^7^ School of Life Science, Anqing Normal University, Anqing, China; ^8^ College of Life Sciences, Capital Normal University, Beijing, China

**Keywords:** LDHB, telomerase, telomere, pancreatic cancer, cell senescence

## Abstract

Telomerase activity is elevated in most cancer cells and is required for telomere length maintenance and immortalization of cancer cells. Glucose metabolic reprogramming is a hallmark of cancer and accompanied with increased expression of key metabolic enzymes. Whether these enzymes influence telomerase activity and cell immortalization remains unclear. In the current study, we screened metabolic enzymes using telomerase activity assay and identified lactate dehydrogenase B (LDHB) as a regulator of telomerase activity. Sodium lactate and sodium pyruvate did not influence telomerase activity, indicating LDHB regulates telomerase activity independent of its metabolism regulating function. Further studies revealed that LDHB directly interacted with TERT and regulated the interaction between TERT and TERC. Additionally, long-term knockdown of LDHB inhibited cancer cell growth and induced cell senescence *in vitro* and *in vivo*. Higher LDHB expression was detected in pancreatic cancer tissues compared with that in adjacent normal tissues and expression of LDHB correlated negatively with prognosis. Thus, we identified LDHB as the first glucose metabolic enzyme contributing to telomerase activity and pancreatic cancer cell immortalization.

## Introduction

Pancreatic cancer is a leading cause of cancer mortality worldwide with over 330,000 new cases and approximately the same number of deaths annually. In contrast to the mortality rates of most other common cancers, the mortality rate of pancreatic cancer has not decreased in the previous decade (1, 2). Immortalization is one of the ten hallmarks of cancer cells, approximately 85% of which are achieved through the activation of telomerase (3, 4). Telomerase contains two essential components responsible for its activity: the TERT protein subunit and the TERC RNA subunit. TERC serves as a template for the addition of repeated DNA fragments (TTAGGG) to the end of telomeres (5). The telomere ends shorten progressively with cell division and triggers cell senescence. While telomerase is inactive in most normal somatic cells, its enzyme activity is elevated in most tumor cells. Previous studies showed that strong telomerase activity was detected in 100% of malignant pancreatic tumors and low or negative telomerase activity was observed in benign tumors (6). Furthermore, telomerase activity has been shown to be related to the progression of pancreatic cancer. Telomerase activity in stage III and IV pancreatic cancer is higher than that in stage I, and the prognosis is worse with the increase of telomerase expression (7). Thus, telomerase is closely related to the development and progression of pancreatic cancer.

Energy metabolism reprogramming is another hallmark of cancer (8). The aberrant glucose metabolism characterized by high glycolysis is associated with dramatically increased bioenergetics, biosynthetic, and redox demands, which are vital to maintain the elevated glucose uptake and lactate production required for rapid cancer cell proliferation, tumor progression, and resistance to chemotherapy and radiation (8–10). Lactate dehydrogenase (LDH) plays key roles in this process. LDHA directly converts pyruvate into lactic acid and is highly expressed in a variety of malignant tumors and is closely related to cancer prognosis (11). An essential role of LDHB in the progression of various cancers has been increasingly reported (12–14). However, the expression and function of LDHB in pancreatic cancer remains largely unclear.

In this study, we investigated the potential relation with metabolic enzymes and telomerase activity in pancreatic cancer and identified that LDHB interacted with TERT and regulated telomerase activity independent of its metabolism regulating function. LDHB promoted the interaction between TERT and TERC. LDHB was required for telomere length maintenance and cell immortalization. These results identified an interaction between glucose metabolism enzymes and telomerase and suggested LDHB as a potential therapeutic target of pancreatic cancer.

## Materials and Methods

### Cell Culture, Plasmids and Reagents

The 293T human embryonic kidney cell line, and PANC-1 and Capan-2 pancreatic cancer cell lines were purchased from the American Type Culture Collection and were previously tested for mycoplasma contamination. Cells were routinely cultured in Dulbecco’s Modified Eagle Medium (Macgene, China) with 10% fetal bovine serum (Hyclone, USA). The FLAG-tagged LDHB eukaryotic expression vector was generated by inserting PCR-amplified fragments of the LDHB gene into pcDNA3 (Invitrogen, USA). Lentiviral shRNA vectors of LDHA and LDHB were constructed by cloning short hairpin RNA fragments into pSIH-H1-Puro (System Biosciences, USA). Target sequences are as follows, LDHA shRNA: 5’- ATCCAGTGGATATCTTGACCTACG -3’; LDHB shRNA: 5’- GGATATACCAACTGGGCTATT -3’. Lentiviruses were produced by co-transfection of HEK293T cells with recombinant lentivirus vectors and pPACK Packaging Plasmid Mix (System Biosciences, USA) using PEI reagent (Polyscience, USA). Lentiviruses were used to infect target cells in accordance with the manufacturers’ instructions. Myc-TERT, Flag-TERT, and GST-TERT plasmids were described previously (15). Sodium pyruvate, Sodium lactate, Methyl pyruvate and IPTG were products from Sigma (USA). 2-DG and AT-101 were purchased from Selleck (USA).

### Transfection and RNA Interference

PEI (Polyscience, USA) was used for plasmid transfection and RNAiMax (Invitrogen, USA) was used for RNA interference in accordance with the manufacturers’ instructions. siRNAs were purchased from Invitrogen (USA). The target sequences are as follows: PKM2 siRNA: 5’-CAUCUACCACUUGCAAUUATT-3’; HK2 siRNA: 5’- GGAGGAUGAAGGUAGAAAUTT-3’; PGK1 siRNA: 5’- CCAAGUCGGUAGUCCUUAUTT-3’; ENO1 siRNA: 5’- CCCAGUGGUGUCUAUCGAATT-3’; LDHA siRNA-1: 5’- GCCGATTCCGGATCTCATT -3’; LDHA siRNA-2: 5’- TCACTGGAGGCCAGGAATT-3’; LDHB siRNA-1: 5’- GGATATACCAACTGGGCTATT -3’; LDHB siRNA-2: 5’- GGCAACAGTTCCAAACAATT-3’.

### Western Blotting

Western blot analysis was performed as described previously (16). Briefly, samples were separated on SDS–polyacrylamide gels and transferred to nitrocellulose membranes. The membranes were incubated with corresponding antibodies. Immunoreactive bands were visualized using the ChemiDoc™ Imaging System (Bio-Rad, USA) with a Super Signal™ West Pico PLUS Chemiluminescent Substrate Kit (Thermo). The primary antibodies included anti-LDHB (ab53292, Abcam, USA), anti-LDHA (66287-1-Ig, Proteintech, China), anti-Flag (A8592, Sigma, USA), anti-Myc (M047-7, MBL, USA), β-actin (sc-47778HRP, Santa Cruz, USA), anti-PKM2 (15822-1-AP, Proteintech, China), anti-HK2 (66974-1-Ig, Proteintech, China), anti-PGK1 (17811-1-AP, Proteintech, China), anti-ENO1 (11204-1-AP, Proteintech, China), anti-TERT for WB (ab32020, Abcam, USA), anti-TERT for IP (abx120550, Abbexa, United Kingdom).

### TRAP Assay

The TRAP assay was performed according to protocol of kit (S7700, Millipore, USA). Briefly, cells were lysed with 1× CHAPS lysis buffer for 30 min on ice, and the lysates were centrifuged at 12,000 rpm for 20 min. The total protein concentration was determined by the Bio-Rad Protein Assay Kit. Indicated amounts of samples were mixed with 2 µl TRAP buffer, 0.4 µl TS primer, 0.4 µl primer mix, 0.4 µl dNTPs, 0.2 µl RNase inhibitor, 0.2 µl Taq DNA polymerase, and 15.4 µl DEPC treated H_2_O, in a total volume of 20 µl. The solution was incubated at 30°C for 30 min, then at 94°C for 30 s to terminate the reaction, followed by 25-30 cycles of 94°C for 30 s, 59°Cfor 30 s and 72°C for 1 min, and a final extemsion at 72°C for 5 min. PCR products were loaded onto a 10% polyacrylamide gel (29:1 acryl/bisacryl) in 0.5×Tris-borate-EDTA (TBE). Gels were run at room temperature for 50min at 200V. The gel was photographed by ChemiDoc™ Imaging System (Bio-Rad). Gray value of the band was analyzed using Scion Image software.

### CRISPR-Cas9-Mediated Knockout of LDHB

LDHB KO PANC-1 cells were generated by CRISPR-Cas9. The single guide RNA sequences targeting LDHB (sgRNA-1: ACTACAGTGATCTTATTGTT, sgRNA-2: TTACCCAAACACCGCGTGAT) were cloned into the lentiCRISPR V2 (Addgene #52961) and packaged into lentivirus in 293T cells, followed by transfection into PANC-1 cells. After selection with puromycin, cells were collected and LDHB expression levels were examined by Western blot.

### Immunofluorescence

Cells grown on glass coverslips were fixed using 4% PFA for 15 min at room temperature, permeabilized using blocking buffer (PBS containing 1% normal goat serum) supplemented with 0.5% TritonX-100 for 20 min on ice, and then washed three times with blocking solution for 10 min each time. The cells were then incubated with rabbit anti-LDHB (14824-1-AP, Proteintech, China) and mouse anti-Myc (M047-A59, MBL, USA) for 2 h at room temperature. After three washed in blocking solution for 10 min each time, the cells were incubated with corresponding secondary antibodies for 1 h at room temperature. The cells were then washed three times with PBS for 10 min each time. Nuclei were counterstained with DAPI. Confocal images were collected using a LSM 780 confocal microscope (Zeiss).

### Co-Immunoprecipitation

Cells were lysed on ice for 30 min in NP40 lysis buffer (25 mM HEPES-KOH at pH 7.5, 1.5 mM MgCl2, 150 mM KCl, 0.5% NP40, 10% glycerol, and 5 mM β-mercaptoethanol supplemented with protease inhibitors). After the lysates were centrifuged at 13000 rpm at 4°C for 30 min, the supernatant was collected, and mixed with anti-flag M2 affinity gel (Sigma, USA) at 4°C for 4 h. The beads were collected by centrifugation at 2000rpm at 4°C for 1 min, washed three times at 4°C with lysis buffer for 10 min each time, mixed with 2× SDS loading buffer, and boiled for approximately 10-20 min. The samples were then analyzed by western blot analysis.

### GST Pull-Down

Purification of GST and GST-TERT fusion proteins was described previously (15). His-LDHB (Fitzgerald) was incubated with GST or GST-TERT at 4 °C for 4 h or overnight. The samples were mixed with 2×SDS loading buffer for SDS-PAGE analysis.

### Quantitative Reverse Transcription-PCR

Total RNA was extracted using TRIzol reagent and equal amounts of RNA were reverse-transcribed using SuperScript II Reverse Transcriptase according to the manufacturer’s instructions (Takara, Japan). qPCR was performed with SYBR-green premix (Takara, Japan) on a CFX96 Real-Time PCR detection system. The primers used for qRT-PCR were as follows: TERC forward, 5’-AAGAGTTGGGCTCTGTCAGC-3’, TERC reverse, 5’-GACTCGCTCCGTTCCTCTTC-3’; TERT forward, 5’-GCGGAAGACAGTGGTGAACT-3’, TERT reverse, 5’-AGCTGGAGTAGTCGCTCTGC-3’; β-actin forward, 5’-ATCACCATTGGCAATGAGCG-3’, β-actin reverse, 5’-TTGAAGGTAGTTTCGTGGAT-3’. β-Actin was used as an internal control. The relative expression was calculated by the comparative Ct method.

### Metabolic Flux Analysis

Seahorse XF96 Extracellular Flux Analyzer (Seahorse Bioscience) was used to analyze extracellular acidification rate (ECAR) and cellular oxygen consumption rate (OCR) of cells as previously described (10). Briefly, PANC-1 cells transfected with siLDHA, siLDHB, shLDHA or shLDHB were harvested and ten thousand cells were then seeded into a Seahorse XF 96 cell culture microplate for 10 hr. After baseline measurements, for ECAR, glucose (10 mM), the oxidative phosphorylation inhibitor oligomycin (1 μM), and the glycolytic inhibitor 2-DG (100 mM) were sequentially injected into each well at the indicated time points. For OCR, oligomycin (1 μM), the reversible inhibitor of oxidative phosphorylation FCCP (p-trifluoromethoxy carbonyl cyanide phenylhydrazone, 1 μM), and the mitochondrial complex I inhibitor rotenone plus the mitochondrial complex III inhibitor antimycin A (1 μM rotenone (Rote)/1 μM antimycin A (AA)) were sequentially injected. Data were analyzed by Seahorse XF-96 Wave software. OCR is reported in pmols/minute and ECAR in mpH/minute. The results were normalized to cell number.

### Measurement of Lactate Production

As previously described (10), one hundred thousand cells were plated into a 12-well plate and incubated in DMEM containing 10% FBS for 10 hr. To measure the secretion of lactate, the media were removed. The cells were washed with DMEM and incubated in DMEM without FBS. After incubation for 1 hr, the supernatant was collected for measurement of lactate production (Biovision). The reaction mixture was incubated for 30 min at room temperature and protected from light. The lactate levels were measured at 450 nm in a microplate reader and normalized with cell number.

### Cell Proliferation

PANC-1 cells were plated in 96-well plates at approximately 3000 cells per well. After the cells adhered to the bottom, 10 μl CCK-8 solution was added to each well. After 2 h, the absorbance at 450 nm of each well was examined by a microplate reader. The growth curve was plotted with the recorded values.

### SA-β-gal Assay

SA-β-gal assay was performed using the SA-β-gal Staining Kit (Beyotime, China), in accordance with the manufacturer’s protocol. Briefly, mouse tumor and human cancer tissues were flash frozen in optimal cutting temperature (OCT) and 5 μm thick sections were cut. Cells or frozen tissue sections were washed once with PBS, and fixed with 0.5% glutaraldehyde in PBS at pH 7.2 for 15 min. After cells were washed in PBS, cells were stained in X-gal solution (100 mM sodium phosphate, 2 mM MgCl2, 150 mM NaCl, 0.01% sodium deoxycholate, 0.02% NP-40, 5 mM potassium ferricyanide, 5 mM potassium ferrocyanide, 1 mg/ml X-gal at pH 6.0) overnight at 37°C. Tissue sections were then stained with eosin.

### qPCR Assay for Average Telomere Length Measurement

The telomere length was measured following the previously published method (17). Genomic DNA was isolated from cells using the KingFisher Flex DNA purification instrument (Thermo Fisher, USA) with MagMAX™ DNA Multi-Sample Ultra 2.0 Kit (ThermoFisher). The primers for telomere PCR were tel1b: 5-CGGTTT(GTTTGG)_5_GTT-3, used at a final concentration of 300 nM, and tel2b: 5-GGCTTG(CCTTAC)_5_CCT-3, used at a final concentration of 300 nM. The primers for single-copy gene (36B4) PCR were 36B4u: 5-CAGCAAGTGGGAAGGTGTAATCC-3, used at a final concentration of 300 nM, and 36B4d: 5-CCCATTCTATCATCAACGGGTACAA-3, used at a final concentration of 500 nM. The 2×Mix (Qiagen, USA) was used in qPCR reaction mixture with 9.2 ng genomic DNA in each tube. qPCR was carried out on CFX-96 qPCR instrument (Bio-Rad, USA). The telomere (T) PCR conditions were 95°C for 10 min and 20 cycles of 95°C for 15 s, 56°C for 1 min. The 36B4 (S) PCR conditions were 95°C for 10min and 30 cycles of 95°C for 15 s, 60°C for 1 min. The relative T/S ratio of each sample was calculated as the relative telomere length. The T/S ratio for each sample was measured twice.

### Animal Experiments

Animal protocols were approved by the Institutional Animal Care and Use Committee of Beijing Institute of Biotechnology. Ten female BALB/c nude mice (4 weeks old, Charles River Laboratories, USA) were randomly divided into two groups, with 5 mice in each group. One group was injected with PANC-1 cells or PANC10.05 cells stably knockdown of LDHB, and the other group was injected with control PANC-1 cells or PANC10.05 cells. A mixture of 100 μl cell suspension (containing 5×10^6^ cells) and 100 μl Matrigel was injected into the back of nude mice. Tumor growth was monitored by vernier caliper measurement every 7 days and the tumor volume was calculated according to the following formula: volume = (longest diameter × shortest diameter^2^)/2. Mice were sacrificed on day 30 after implantation. Subcutaneous tumors were dissected and isolated, and tumor size was measured. The tumor tissues were divided into four parts. The first sample was used for qRT-PCR to detect the expression of LDHB. The second tissue sample was paraffin-embedded and sectioned for immunohistochemistry to detect LDHB expression and used in quantitative fluorescence *in-Situ* hybridization (Q-FISH) to detect telomere length. The third sample was frozen and subjected to SA-β-gal staining to detect cell senescence. The fourth tumor sample was used for TRAP assay to detect telomerase activity.

### Q-FISH

The paraffin slides were incubated in 10 mM sodium citrate (pH 6.5) at 88°C for 10 min, rinsed with PBS (pH 7.2) at room temperature for 1 min, dried with 25%, 50% and 95% ethanol, and treated with 1% pepsin solution at 37°C for 2 min. Each slide was incubated with 80 μl 10 mg/ml RNase A solution (NanoMagBio, China), covered with cover glass, and placed in the heating block at 37°C for 2 h. The cover glasses were removed and the slides were washed with PBS for 1 min. The slides were then briefly immersed in 25%, 50% and 95% ethanol and air dried. Next, each sample was hybridized with 100 μl telomere probe (TelC-Alexa488, PANAGENE) overnight in the dark at room temperature (at least 16 h). After hybridization, the slides were placed in a Coplin flask with 70% formamide buffer and rinsed for 15 min. The slides were rinsed with fresh formamide buffer for four times, 15 min each time followed by washing with Tween 20 buffer four times for 5 min each time at room temperature. Nuclei were counterstained with 80μl DAPI for 5 min at room temperature. The slides were observed under a fluorescence microscope as soon as possible or stored in a closed box at -20°C. Fluorescence intensity was then quantified using the ImageJ (http://rsb.info.nih.gov/ij/) plugin Telometer (http://demarzolab.pathology.jhmi.edu/telometer/index.html).

### Clinical Samples and Immunohistochemistry

Paraffin specimens of pancreatic cancer tissue were obtained from surgically removed tissues of inpatients in the Faculty of Hepato-Pancreato-Biliary Surgery, Chinese PLA General Hospital. The ethics of the study was approved by the Institutional Review Board of Chinese PLA General Hospital (the reference number is S2016-098-01). IHC of formalin-fixed paraffin-embedded samples was performed as described previously (18). Rabbit anti-LDHB (ab32020, Abcam, USA) was used at a dilution of 1:150. The expression of LDHB was determined by calculating the expression score which was generated by multiplying the score for intensity of the staining (no staining=0; weak staining=1; moderate staining=2; strong staining=3) by the percentage of stained cells (0%-100%). Expression score above 1.6 was determined as high expression and below 1.6 was set as low expression.

### Statistical Analysis

Comparisons between two groups were performed using Student’s t-test. The data are presented as the means ± standard deviation (SD). Estimation of overall survival was performed using the Gehan-Breslow-Wilcoxon test. Statistical calculations were performed using Prism, p <0.05 was considered statistically significant.

## Results

### LDHB Regulates Telomerase Activity in Pancreatic Cancer Cells Independent of Metabolic Regulation Activity

**Figure 1:**
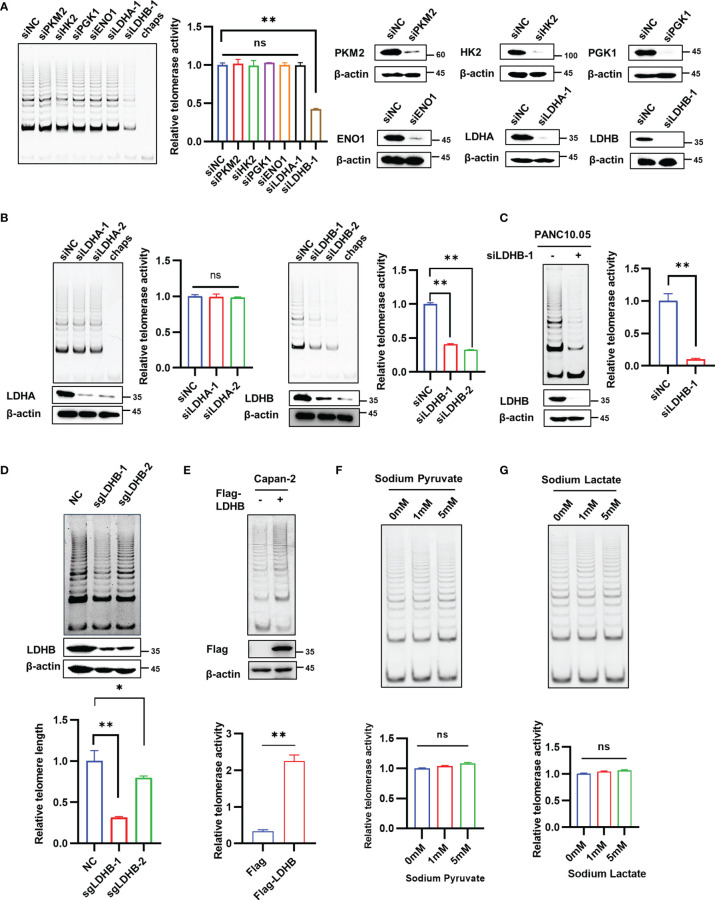
LDHB regulates telomerase activity in pancreatic cancer cells independent of its regulation on metabolic pathways. **(A)** PANC-1 cells were transfected with indicated siRNAs for 2 days and TRAP assay was performed to detect telomerase activity. Western blot assay was performed to detect the knockdown effect of siRNA. **(B)** The telomerase activity of knockdown of LDHA or LDHB with two siRNAs were detected by TRAP assay in PANC-1 cells. The expression of LDHA or LDHB were detected by western blot with the indicated antibodies. **(C)** The telomerase activity of knockdown of LDHB was detected by TRAP assay in PANC10.05 cells. **(D)** PANC-1 cells were stably transfected with LDHB sgRNAs and telomerase activity was examined. **(E)** TRAP assay in Capan-2 cells transfected with Flag or Flag-LDHB. The expression of Flag-LDHB was detected by western blot. **(F, G)** PANC-1 cells were treated with indicated concentrations of sodium pyruvate **(F)** or sodium lactate **(G)** for 48 h and telomerase activity was detected. Data are shown as mean ± SD of three independent experiments. (*P<0.05, **P<0.01, ns means no statistically significant).

To determine whether enzymes involved in glucose metabolism regulate telomerase activity, we performed a small-scale screen with siRNAs targeting genes encoding well-known metabolic enzymes, including PKM2, HK2, PGK1, ENO1, LDHA and LDHB in PANC-1 cells. We confirmed successful knockdown of each gene (**Figure 1A**). The results showed that knockdown of LDHB, but not other factors, significantly decreased telomerase activity (**Figure 1A**). Since LDHA and LDHB share a high similarity, we confirmed the specificity of antibodies using cell lysates transfected with LDHA siRNA and LDHB siRNA (**Figure S1A**). We found that knockdown of LDHA did not affect the expression of LDHB, and vice versa (**Figure S1A**). We next confirmed the results with two siRNAs target LDHA and LDHB, respectively. While the telomerase activity of PANC-1 cells decreased after LDHB knockdown, there was no significant changes in telomerase activity after LDHA knockdown (**Figure 1B**). The effect of LDHB siRNA on telomerase activity was also examined in PANC10.05 cells (**Figure 1C**). Additionally, CRISPR-Cas9 system was used to exclude off-target effect. PANC-1 cell expressing LDHB sgRNAs have a decreased telomerase activity (**Figure 1D**), which is consistent with the results of siRNA. To examine the effect of overexpression of LDHB on telomerase activity, we transfected PANC-1 cells with LDHB expression plasmid with or without Flag tag. Both vectors increased the telomerase activity in PANC-1 cells (**Figure S1B**). Flag-LDHB was also transfected into Capan-2 cells which does not express endogenous LDHB (https://www.proteinatlas.org/ENSG00000111716-LDHB/cell+line). The results showed that overexpression of LDHB promote the telomerase activity in Capan-2 cells (**Figure 1E**). These results suggest that LDHB promotes telomerase activity in pancreatic cancer cells.

LDHB is a member of the lactate dehydrogenase family and mediates the conversion between lactic acid and pyruvate. To determine whether LDHB regulated telomerase activity through its effect on metabolic pathways. Wild type PANC-1 cells and PANC-1 cells transfected with LDHB siRNA were treated with different concentrations of sodium pyruvate (**Figure 1F** and **Figure S1C**), sodium lactate (**Figure 1G** and **Figure S1D**) or methyl pyruvate (**Figure S1E**), and the telomerase activity in each group was detected 2 days later. The results indicate that addition of sodium pyruvate, sodium lactate or methyl pyruvate had no impact on telomerase activity. In addition, we used 2-DG and AT-101 to inhibit glycolysis to detect whether targeting glycolysis regulate telomerase activity. Neither 2-DG nor AT-101 regulate telomerase activity in PANC-1 cells (**Figures S1F, G**). These findings indicate that LDHB regulates telomerase activity independent of its effect on metabolic pathways.

### LDHB Interacts With TERT and Regulates Interaction Between TERT and TERC

**Figure 2:**
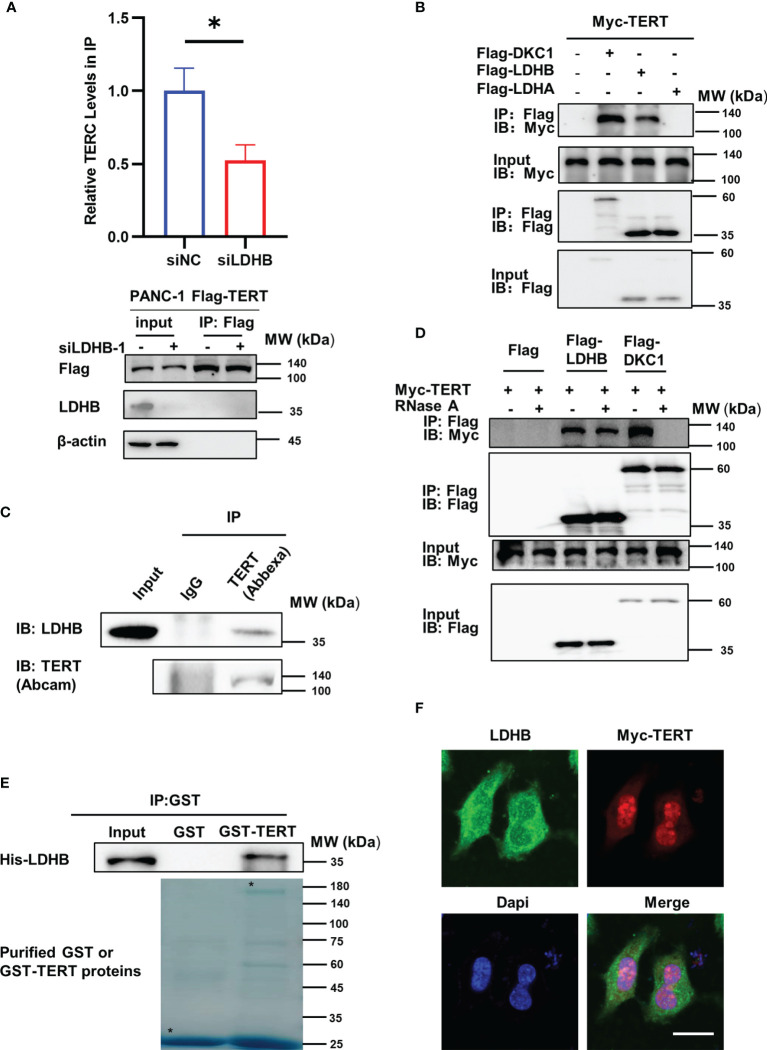
LDHB interacts with TERT and regulates interaction between TERT and TERC. **(A)** PANC-1 Flag-TERT cells were transiently transfected with LDHB siRNA-1 or control siRNA and immunoprecipitated with anti-Flag followed by immunoblot analysis for Flag-TERT, LDHB and β-actin expression. Relative TERC levels in IP lysates were determined by qRT-PCR. Data are representative of three independent experiments (mean ± SD of two technical replicates. * P < 0.05). **(B)** 293T cells were transiently transfected with Myc-TERT and Flag-LDHB, Flag-LDHA or Flag-DKC1 (as a positive control), and lysates were immunoprecipitated with anti-Flag. Flag antibody was used for immunoblotting (IB) to detect LDHB, LDHA and DKC1, and Myc antibody was used to detect TERT expression. **(C)** PANC-1 cell lysates were immunoprecipitated with IgG or anti-TERT (Abbexa) and immunoblotted with indicated antibodies. **(D)** HEK293T cells were transiently transfected with Myc-TERT and Flag-LDHB or Flag-DKC1 plasmids. Lysates were treated with or without RNase A (0.1mg/ml). Anti-Flag was used for IP. LDHB and DKC1 were detected by IB with Flag antibody, and TERT expression was detected by Myc antibody. **(E)** Glutathione-Sepharose beads bound with GST-TERT or GST were incubated with purified His-tagged LDHB. After washing the beads, the bound proteins were examined by immunoblot with anti-His antibody. Purified proteins were examined by Coomassie brilliant blue staining after SDS-PAGE. Asterisks indicate the positions of the expected full-length fusion proteins. **(F)** PANC-1 cells were transiently transfected with Myc-TERT plasmid. The localization of LDHB and TERT were detected by immunofluorescence. Scale bar, 20μm.

Telomerase mainly comprises TERT and TERC, which could reconstitute telomerase activity *in vitro* (19). To explore the mechanism of the regulation of LDHB on telomerase activity, we first examined whether LDHB regulated the expression of TERT and TERC in PANC-1 cells. Because of the small amount of endogenous TERT (20), detecting endogenous TERT protein levels is challenging. Thus, we evaluated whether LDHB regulates endogenous TERT mRNA levels in PANC-1 cells and exogenous TERT protein levels in PANC-1 cells stably transfected with Flag-TERT (PANC-1 FT cells). Knockdown of LDHB did not regulate TERT mRNA levels and exogenous TERT protein levels, but it resulted in increased levels of TERC (**Figures S2A, B**). Elevated TERC will increase telomerase activity, which is inconsistent with our findings of telomerase activity. These results indicate that other mechanisms contribute to the LDHB mediated changes of telomerase activity. We next evaluated whether LDHB regulates the interaction between TERT and TERC, which is considered as the minimal complex harboring telomerase activity. Lysates from PANC-1 FT cells transfected with LDHB siRNA were subjected to immunoprecipitation with Flag beads to immunoprecipitated Flag-TERT complexes, which were then examined by western blot for the detection of TERT and RNA extraction for the detection of TERC. The results showed that the interaction of TERT and TERC decreased after knockdown of LDHB (**Figure 2A**), which suggested that LDHB regulates telomerase activity in pancreatic cancer cells by affecting the assembly of TERT and TERC. We next examined the interaction between LDHB and telomerase by transfecting 293T cells with Myc-TERT and Flag-LDHA or Flag-LDHB and performing immunoprecipitation assays. Cells transfected with Myc-TERT and Flag-DKC1 were included as positive control. The results showed that TERT interacted with LDHB, but not LDHA (**Figure 2B**). To detect endogenous interaction between TERT and LDHB, endo-IP assay was performed using TERT antibody (Abbexa) as previously described (15). IP lysates were used to detect LDHB and TERT proteins with indicated antibodies. The result illustrates that endogenous LDHB interacted with endogenous TERT (**Figure 2C**). To determine whether the interaction between LDHB and TERT was mediated by TERC, we treated cell lysates with RNase A to remove TERC, followed by immunoprecipitation. RNase A treatment disrupted the interaction of TERT and DKC1, which is mediated by TERC (21, 22). However, the interaction between TERT and LDHB was not affected by RNase A (**Figure 2D**), suggesting that LDHB interacted with TERT independent of TERC. We next performed GST pull-down experiments using purified His-LDHB, GST and GST-TERT proteins. The results showed that the His-LDHB protein interacted with purified GST-TERT (**Figure 2E**), indicating that LDHB directly interacts with TERT. We also transfected Myc-TERT plasmid into PANC-1 cells and examined the localization of LDHB and TERT by immunofluorescence staining. LDHB was mostly cytoplasmically localized, but some LDHB co-localized with Myc-TERT in the nucleus (**Figure 2F**).

### LDHB Regulates Telomere Length and Cell Senescence in Pancreatic Cancer Cells

**Figure 3:**
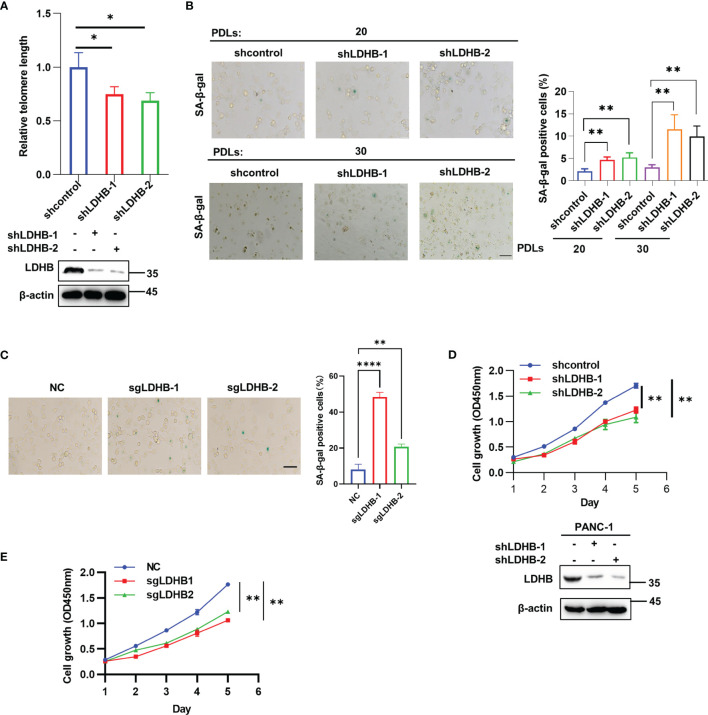
LDHB regulates telomere length and cell senescence in pancreatic cancer cells. **(A)** The telomere length of two PANC-1 single clone cells stably expressing shRNA of LDHB at 20 population doublings (PDLs) was detected by qPCR. The expression of LDHB were detected by western blot analysis. **(B)** Proportion of senescent cells of monoclonal PANC-1 cells stably expressing shRNA of LDHB at 20 PDLs and 30 PDLs were detected by SA-β-Gal staining. The percentage of senescence positive cells in each group from five randomly chosen fields was calculated. Scale bars, 50 μm. **(C)** PANC-1 cells stably expression LDHB sgRNAs were cultured for 25 PDLs and SA-β-Gal staining was performed. Scale bars, 50 μm. **(D)** Proliferation of PANC-1 shLDHB monoclonal cells at 20 PDLs was detected by CCK-8 assay. The expression of LDHB was detected by western blot analysis. **(E)** PANC-1 cells stably expression LDHB sgRNAs were cultured for 25 PDLs and CCK-8 assay was performed. Data are representative of three independent experiments (mean ± SD of three biological replicates **(A, D, E)** or three technical replicates **(B, C)**. * P< 0.05, ** P< 0.01, **** P< 0.0001.

Telomerase is required for telomere length maintenance and prevents cells from senescence. Our results showed that knockdown of LDHB inhibited telomerase activity, and therefore we examined whether telomere length and cell senescence were regulated by LDHB. Relative telomere length was measured in pancreatic cancer cells with transient knockdown of LDHB and long-term knockdown of LDHB by qPCR. LDHB siRNA transfection did not affect telomere length at 3 days post-transfection (**Figure S3A**). Then, we constructed two PANC-1 single clone cells stably expressing LDHB shRNA (**Figure 3A**). LDHB shRNA reduced telomerase activity which was rescued by re-expression of LDHB (**Figure S3B**). Importantly, these cells showed reduced telomere length at approximately 20 population doubling levels (PDLs) (**Figure 3A**). In contrast, stable knockdown of LDHA had no impact on telomere length (**Figure S3C**). Next, we detected the effect of short-term and long-term inhibition of LDHA and LDHB on glycolytic phenotype in PANC-1 cells. We measured extracellular acidification rates (ECARs) by means of the Seahorse XF96 extracellular flux analyzer. Upon addition of glucose, both short-term and long-term inhibition of LDHA and LDHB yielded less ECARs compared to control cells. The ECAR values following oligomycin addition increased in both control cells and LDHB knockdown cells and the values in LDHA and LDHB knockdown cells reduced significantly compared to that in control cells (**Figures S4A, B**). At the same time, oxygen consumption rates (OCRs) were measured. Both short-term and long-term inhibition of LDHA and LDHB displayed an increased basic respiration and glucose oxidation levels, as well as maximal respiration following FCCP addition (**Figures S4C, D**). In addition, both short-term and long-term knockdown of LDHA and LDHB reduced lactate production (**Figures S4E, F**). These results suggest that although the metabolic effects of knocking down LDHA and LDHB are similar (reduced glycolysis and increased mitochondrial respiration), the effects on telomerase activity are different. It is further support the point that the effects of LDHB in telomerase activity are not related to the effects on metabolism.

We next examined the effect of LDHB on cell senescence. Cellular senescence induced by telomere shortening is characterized by typical alterations in cell morphology (flattened cells with enlarged cytoplasm) and elevated senescence-associated β-galactosidase (SA-β-gal) activity (23). SA-β-gal positive cells increased in single clone cells expressing LDHB shRNA at 20 and 30 PDLs. Notably, the ratio of senescent cells increased gradually with increasing PDLs (**Figure 3B**). Re-expression of LDHB partially rescued senescence phenotype in LDHB stable knockdown cells (**Figure S5A**). PANC-1 cells stably expressing LDHB sgRNAs also displayed increased cell senescence (**Figure 3C**). In contrast, stable knockdown of LDHA did not induce cell senescence (**Figure S5B**). Senescent cells also lose the ability to proliferate. CCK-8 assays showed that transient knockdown of LDHB did not inhibit PANC-1 cell proliferation (**Figure S5C**). However, stable knockdown of LDHB inhibited cell proliferation at 25PDLs (**Figure 3D**). Re-expression LDHB rescued cell growth defect (**Figure S5D**). Importantly, knockout LDHB by sgRNA also inhibit cell proliferation (**Figure 3E**). These results suggested that LDHB is required for telomere length maintenance and prevented cells from senescence. We speculate that the functional discrepancy of transient and long-term knockdown of LDHB is mainly because that telomeres shorten gradually with cell division and trigger senescence when they become too short to maintain telomere integrity (24, 25).

### Knockdown of LDHB Inhibits Tumor Growth in Nude Mice

**Figure 4:**
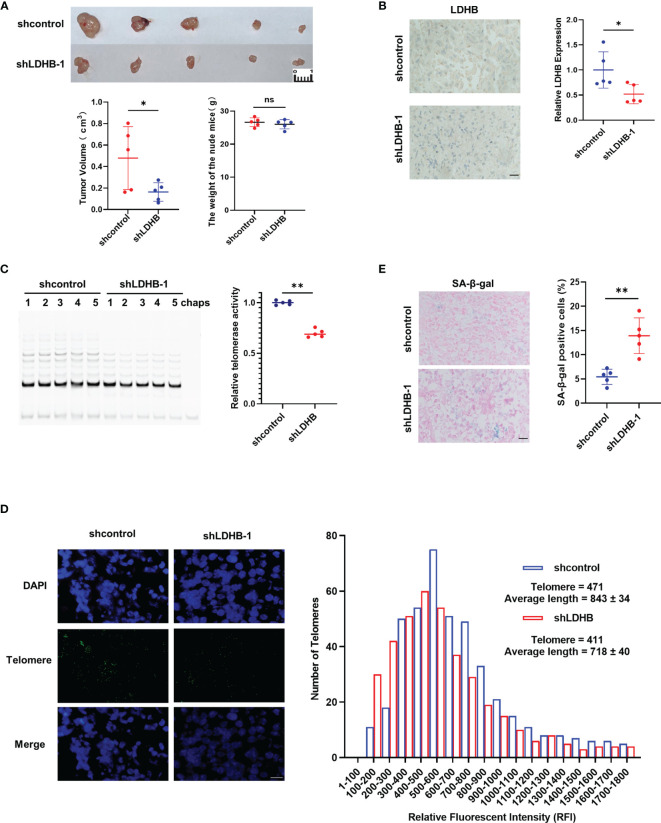
Knockdown of LDHB inhibit the proliferation of PANC-1 cells in nude mice. **(A)** PANC1 cells stably expressing shcontrol or shLDHB were injected subcutaneously in nude mice (n=5 for each group). Tumor value and mice bodyweight were measured 1 month later. (*P <0.05). **(B)** The expression of LDHB in tumors of two groups was detected by immunohistochemistry (*P<0.05). Scale bar, 50 μm. **(C)** Telomerase activity in tumors of each mice were detected by TRAP (**P < 0.01). **(D)** Telomere length was evaluated in paraffin embedded sections of tumor tissue using Q-FISH. Generated histograms of individual telomere length distributions [shorter to longer, lower to higher relative fluorescent intensity (RFI)] for shcontrol (blue) and shLDHB (red). The average telomere length is shown as the average RFI ± SD. Scale bar, 50 μm. **(E)** Representative frozen tumor sections from mice were subjected to SA-β-gal staining followed by eosin staining. The right panel shows the percentage of senescence positive cells of each sample (**P<0.01). Scale bar, 50 μm. ns means no statistically significant

Our *in vitro* experiments showed that knockdown of LDHB inhibited the growth of pancreatic cancer cells. Next, we evaluated whether LDHB exerted similar effects on cell proliferation *in vivo* using nude mice. Nude mice were subcutaneously injected with PANC-1 cells (**Figure 4**) or PANC10.05 cells (**Figure S6**) which stably transfected with control shRNA or LDHB shRNA and monitored for one month. The bodyweight of mice and the tumor volume were measured (**Figures 4A**, **S6A**). The results indicated that knockdown of LDHB significantly inhibited tumor growth. We confirmed decreased LDHB expression in tumors from mice injected with cells expressing LDHB shRNA and further found that telomerase activity was also decreased in these tumors (**Figures 4B, C**, **S6B, C**). Telomere length detected by Q-FISH was also reduced by knockdown of LDHB (**Figures 4D**, **S6D**), which was consistent with the findings *in vitro*. Tumors expressing LDHB shRNA also displayed elevated proportion of SA-β-gal positive cells (**Figures 4E**, **S6E**), which indicated cell senescence.

### LDHB Is Elevated in Pancreatic Cancer

**Figure 5:**
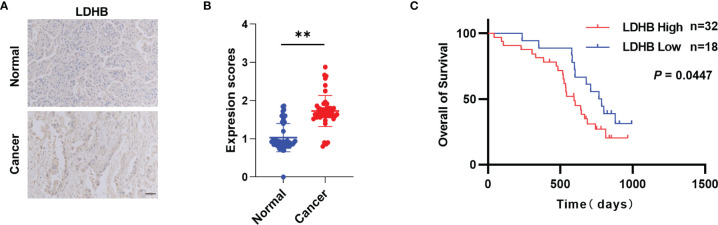
LDHB is elevated in pancreatic cancer. **(A)** Representative immunohistochemical staining of LDHB in 50 pairs of pancreatic cancer tissues and adjacent normal pancreas tissues. Scale bar: 50 μm. **(B)** Expression scores of LDHB between normal and cancer tissues were compared by paired t-test (**P < 0.01). **(C)** Kaplan-Meier survival curves for overall survival of 50 pancreatic cancer patients stratified into high and low LDHB expression groups.

To explore the clinical significance of LDHB in pancreatic cancer, we performed IHC to determine LDHB protein expression in 50 pairs of pancreatic cancer and matched adjacent normal tissues. Compared with adjacent normal tissues, pancreatic cancer tissues expressed higher levels of LDHB (**Figures 5A, B**). These findings suggest that LDHB expression is elevated in pancreatic cancer. We next evaluated the correlation of LDHB expression with clinical prognosis. High expression of LDHB correlated negatively with the overall survival of pancreatic cancer patients (**Figure 5C**). In addition, we analyzed the correlation of relative transcriptional expression of LDHB and overall survival (OS) and disease-free survival (DFS) from Gene Expression GEPIA (26) and generate Kaplan-Meier plots (**Figures S7A, B**). Although no significant correlation was detected (p>0.05), there is a tendency that LDHB negatively correlate with the DFS rate.

## Discussion

Our work identified LDHB as a novel regulator of telomerase activity which is required for telomere length maintenance and prevents cancer cells from senescence. Mechanistic studies revealed that LDHB bound to TERT directly and regulated the interaction of TERT and TERC. LDHB expression was elevated in pancreatic cancer tissues and correlated negatively with overall survival of patients. To the best of our knowledge, LDHB is the first identified glucose metabolic enzyme that interacts with telomerase and regulates cell senescence.

LDHB is a member of the LDH family and LDH is a tetrameric enzyme composed of LDHA and LDHB subunits. LDHA and LDHB show different distributions among different organs and exert different functions. LDHA catalyzes the reduction of pyruvate to lactate, whereas LDHB has higher activity in the oxidation of lactate to pyruvate. In muscle or liver, most LDH complexes are composed of four LDHA subunits, and preferably catalyze the reduction of pyruvate to lactate. However, in the heart and brain, LDHB is the dominant subunit of LDH complex (27). Here we identified a non-classical function of LDHB. We demonstrated that LDHB interacts with TERT and regulates telomerase activity independent of its control of metabolic pathway. Although LDHB is mainly distributed in mitochondria, nuclear localization of LDHB has been detected by transmission electron microscopy (28), which is consistent with our findings (**Figure 2F**). GST pull-down assay using purified proteins further revealed a direct interaction of LDHB and TERT (**Figure 2E**). It is unclear how LDHB regulates the interaction between TERT and TERC, as well as TERC levels, which merit future investigations. Notably, this is not the first identified non-classical function of LDHB. A previous study revealed that LDHB is a key contributor to lysosomal activity and autophagy in both oxidative cancer cells and glycolytic cancer cells through regulation of the conversion of NAD+ and NADH (12).

The activation of telomerase in cancer cells is a complicated process involving at least three steps: TERT expression, telomerase assembly and telomere elongation. TERT expression is downregulated in most of somatic cells upon differentiation due to epigenetic silencing with a few exceptions but its expression reactivated in most of the cancer cells, partially because of core promoter mutations (29, 30). Telomerase assembly and telomere elongation were not considered critical determinants for the differences of telomerase activity between normal cells and cancer cells and the function of cancer-associated proteins in these steps are largely unknown. Although the whole enzyme of telomerase is a large complex mainly comprised of TERT, TERC, DKC1, TCAB1, NHP2, NOP10 and GAR1 (31), previous studies showed that TERT and TERC could reconstitute telomerase activity *in vitro*. This suggest the interaction of TERT and TERC is the key step of telomerase assembly. Accessory proteins, such as Pontin, Reptin, HSP90 and p23 (32, 33), were reported to regulate telomerase assembly. However, the direct interaction between these proteins and TERT was not examined because the lack of purified TERT protein. Our previous study successfully constructed coding sequence optimized GST-TERT and identified that PES1, which expression elevated in many cancers, promoted assembly of TERT and TERC in breast cancer cells (15). In this manuscript, we detected a direct interaction of TERT and LDHB using purified proteins (**Figure 2E**).

Telomerase has long been considered as an attractive target for cancer therapy, and telomerase inhibitors have been shown to cause telomerase inhibition and subsequent telomere shortening and senescence in cancer cells (34–36). The concept of the Reverse Warburg effect has led to the idea for a new anti-cancer treatment modality by preventing the generation and transport of lactate (37, 38). LDHA inhibitors have been widely explored and proven to suppress tumor growth among different cancers (39–41). Although LDHB exerts a reverse function which convert lactate to pyruvate under physiological conditions, a recent study revealed that Aurora-A mediated phosphorylation of LDHB S162 markedly enhances its catalytic activity to convert pyruvate to lactate and promotes glycolysis and tumor progression (13). Increasing evidence suggests that LDHB is critical for malignant progression in triple-negative breast cancer (42), K-Ras amplified lung cancer (14) and colon cancer (43). Furthermore, LDHB expression is elevated in different types of tumors, such as triple-negative breast cancer (42), thyroid cancer (44), laryngeal squamous cell carcinoma (45), lung adenocarcinoma (14), bladder transitional cell carcinoma (46), osteosarcoma (47), testicular germ cell tumor (48) and colorectal cancer (49). Our findings indicated that LDHB is elevated in pancreatic cancers and correlated negatively with the overall survival of pancreatic cancer patients, which is consistent with the RNA-seq data (**Figure S7**) and reported results (50). However, contradictory results have been reported that LDHB expression was reduced in pancreatic cancer tissues compared to normal tissues and inhibition of LDHB promotes pancreatic cancer progression under hypoxia *via* inducing glycolytic phenotype (51). The discrepancy of LDHB on glycolysis and tumor progression may result from different cell lines were used and different oxygen concentration. In addition, we mainly focused on long-term effect of knockdown of LDHB and found that knockdown of LDHB results in gradually telomere attrition, cell senescence and inhibition of tumor growth, which was not detected under short-term knockdown of LDHB. It is interesting to further examine the relationship of LDHB expression, telomerase activity and cell senescence in tumor tissues, which fresh tumor tissues is required. Therefore, we speculate that targeting LDHB may present a new avenue for pancreatic cancer treatment.

## Data Availability Statement

The original contributions presented in the study are included in the article/**Supplementary Msaterial**. Further inquiries can be directed to the corresponding authors.

## Ethics Statement

The studies involving human participants were reviewed and approved by Institutional Review Board of Chinese PLA General Hospital. The patients/participants provided their written informed consent to participate in this study. The animal study was reviewed and approved by Institutional Animal Care and Use Committee of Beijing Institute of Biotechnology.

## Author Contributions

LC, QY and RL conceived the study, designed the experiments and analyzed the data. RW and JL designed and performed the experiments aided by CZ, XG, LQ and RJ, BQ performed IHC staining and analyzed related data aided by SX and XZ, RW and LC drafted the manuscript.

## Funding

LC was supported by the National Natural Science Foundation (82072717) and National Key Research and Development Program of China (2022YFC3600100). QY was supported by National Natural Science Foundation (81630067 and 81930078). RW was supported by Project of the eighth medical center of PLA General Hospital (2021MS003).

## Conflict of Interest

The authors declare that the research was conducted in the absence of any commercial or financial relationships that could be construed as a potential conflict of interest.

## Publisher’s Note

All claims expressed in this article are solely those of the authors and do not necessarily represent those of their affiliated organizations, or those of the publisher, the editors and the reviewers. Any product that may be evaluated in this article, or claim that may be made by its manufacturer, is not guaranteed or endorsed by the publisher.
