# Supplementary figures and images for: Lactate Dehydrogenase B Is Required for Pancreatic Cancer Cell Immortalization Through Activation of Telomerase Activity
Source: Front Oncol. 2022 May 20;12:821620. doi: 10.3389/fonc.2022.821620 (PMC9163669; doi:10.3389/fonc.2022.821620)

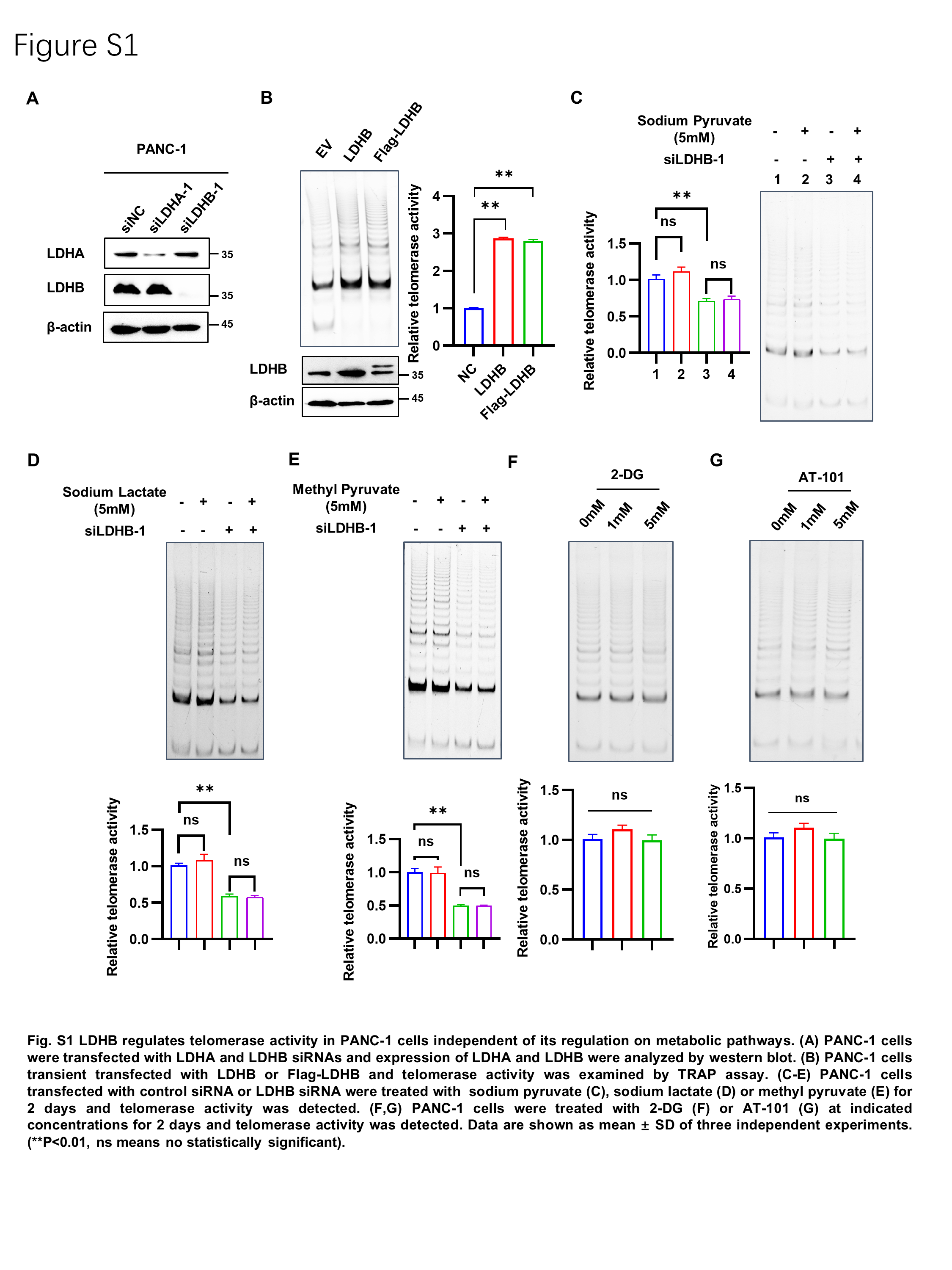

Supplement: Supplementary file 1 [file Image_1.tif]

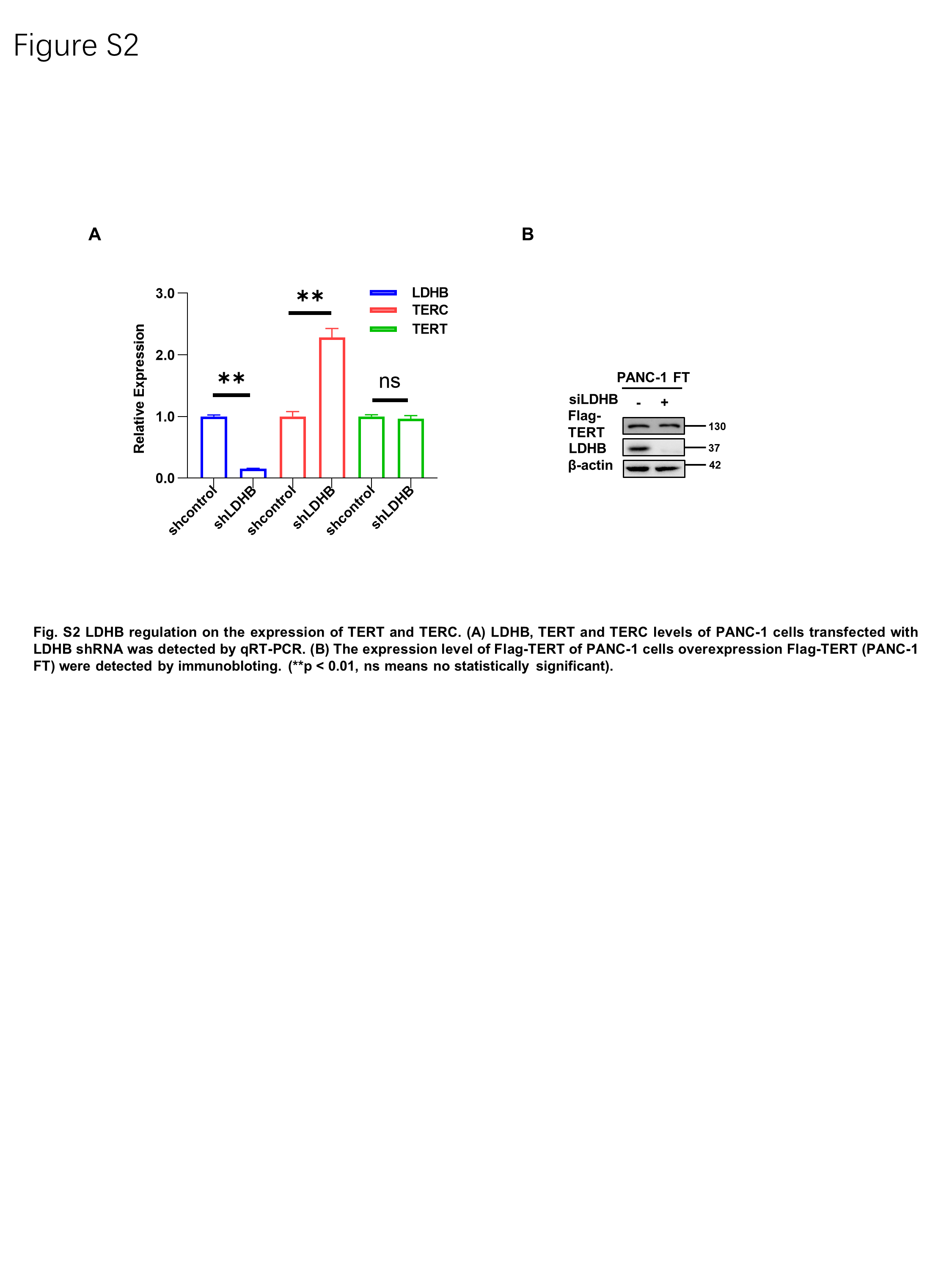

Supplement: Supplementary file 2 [file Image_2.tif]

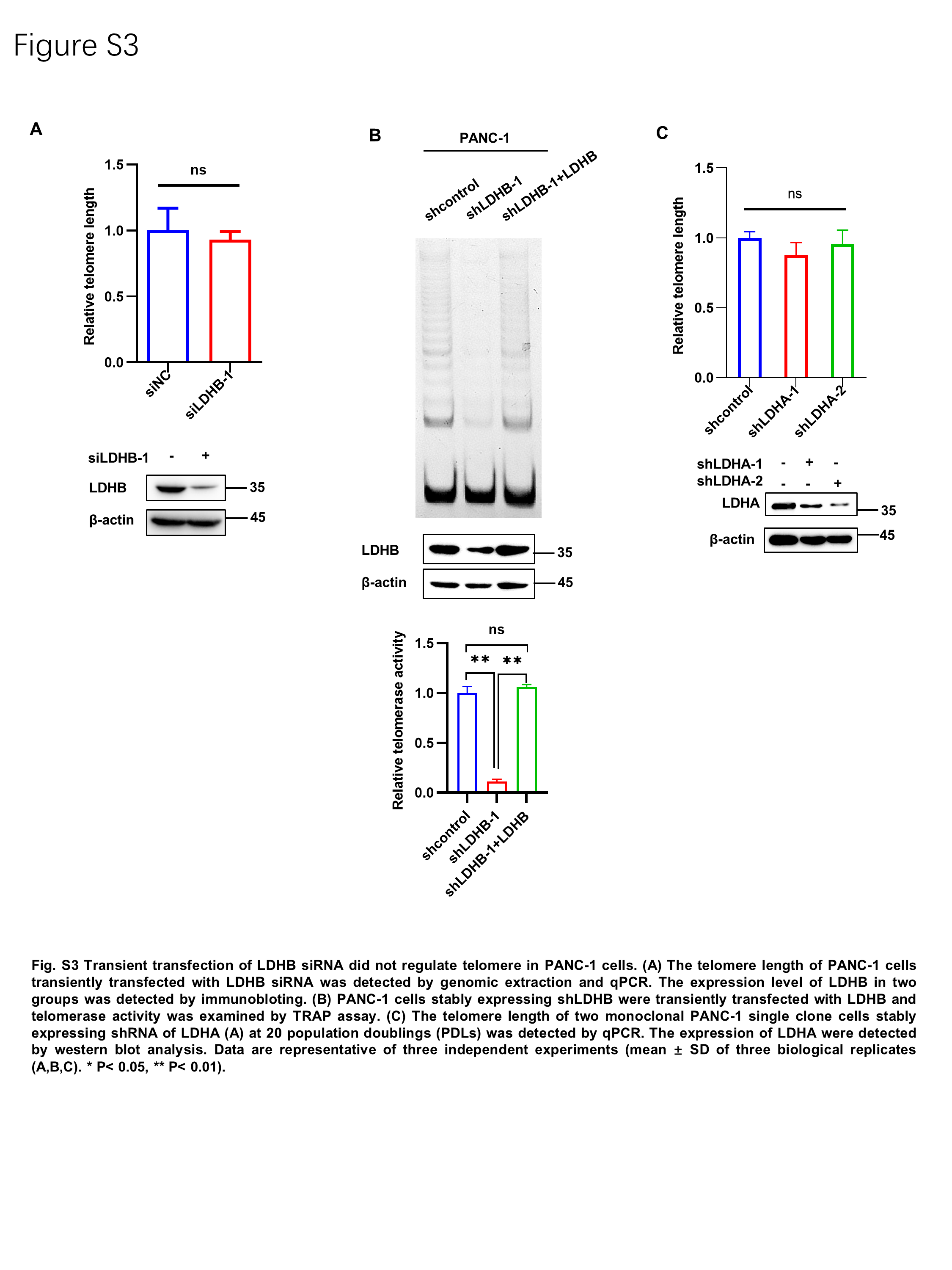

Supplement: Supplementary file 3 [file Image_3.tif]

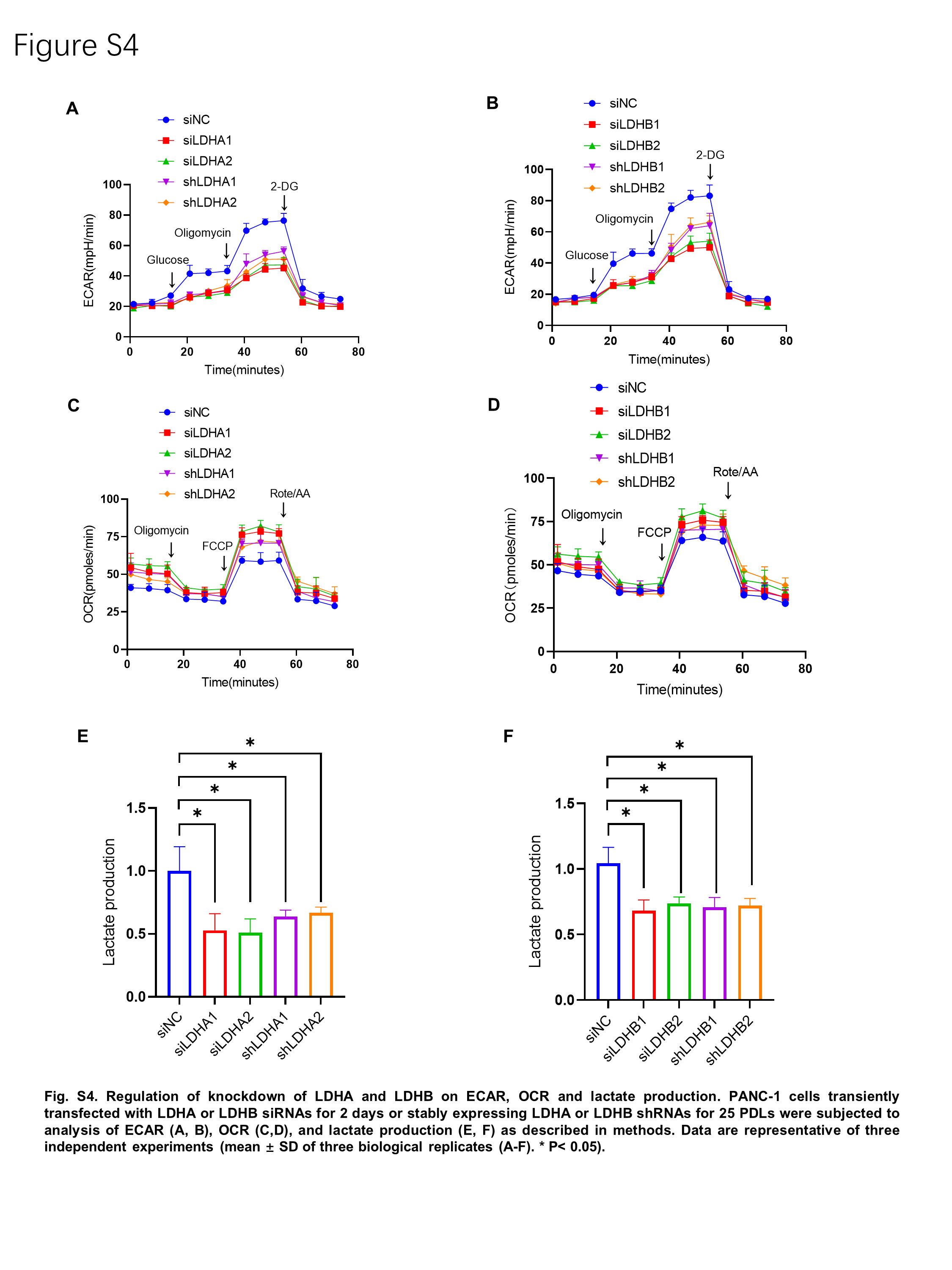

Supplement: Supplementary file 4 [file Image_4.tif]

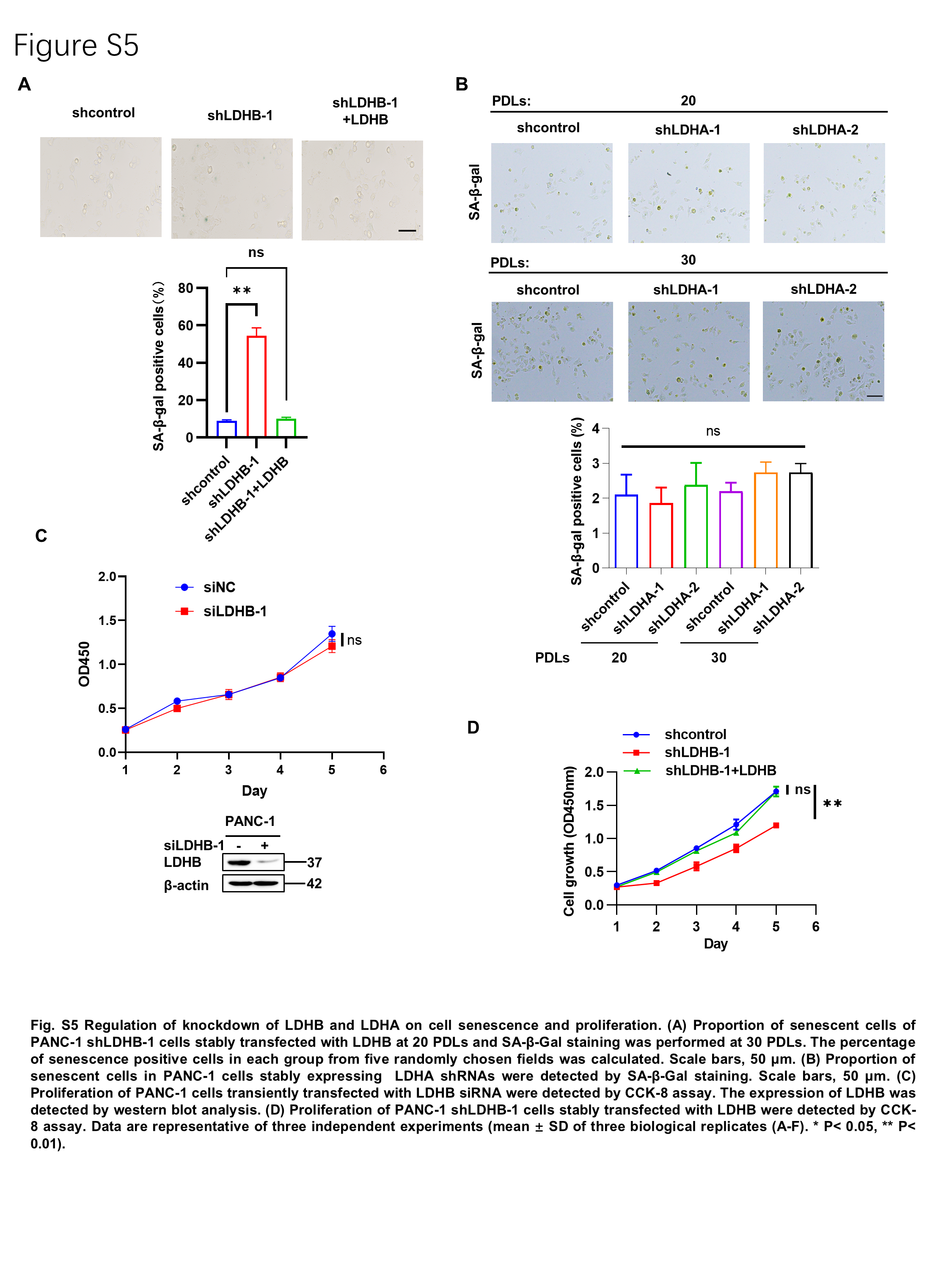

Supplement: Supplementary file 5 [file Image_5.tif]

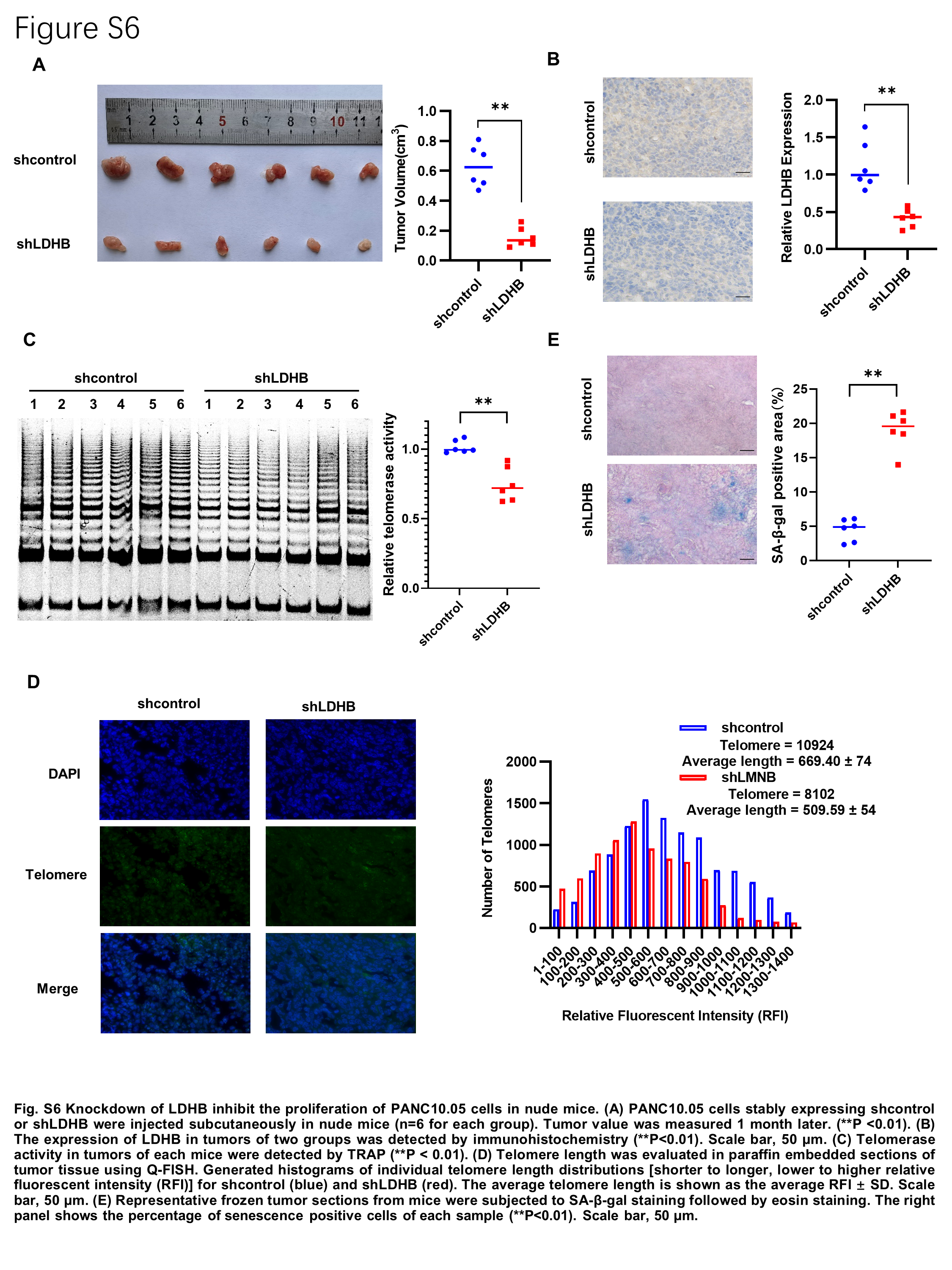

Supplement: Supplementary file 6 [file Image_6.tif]

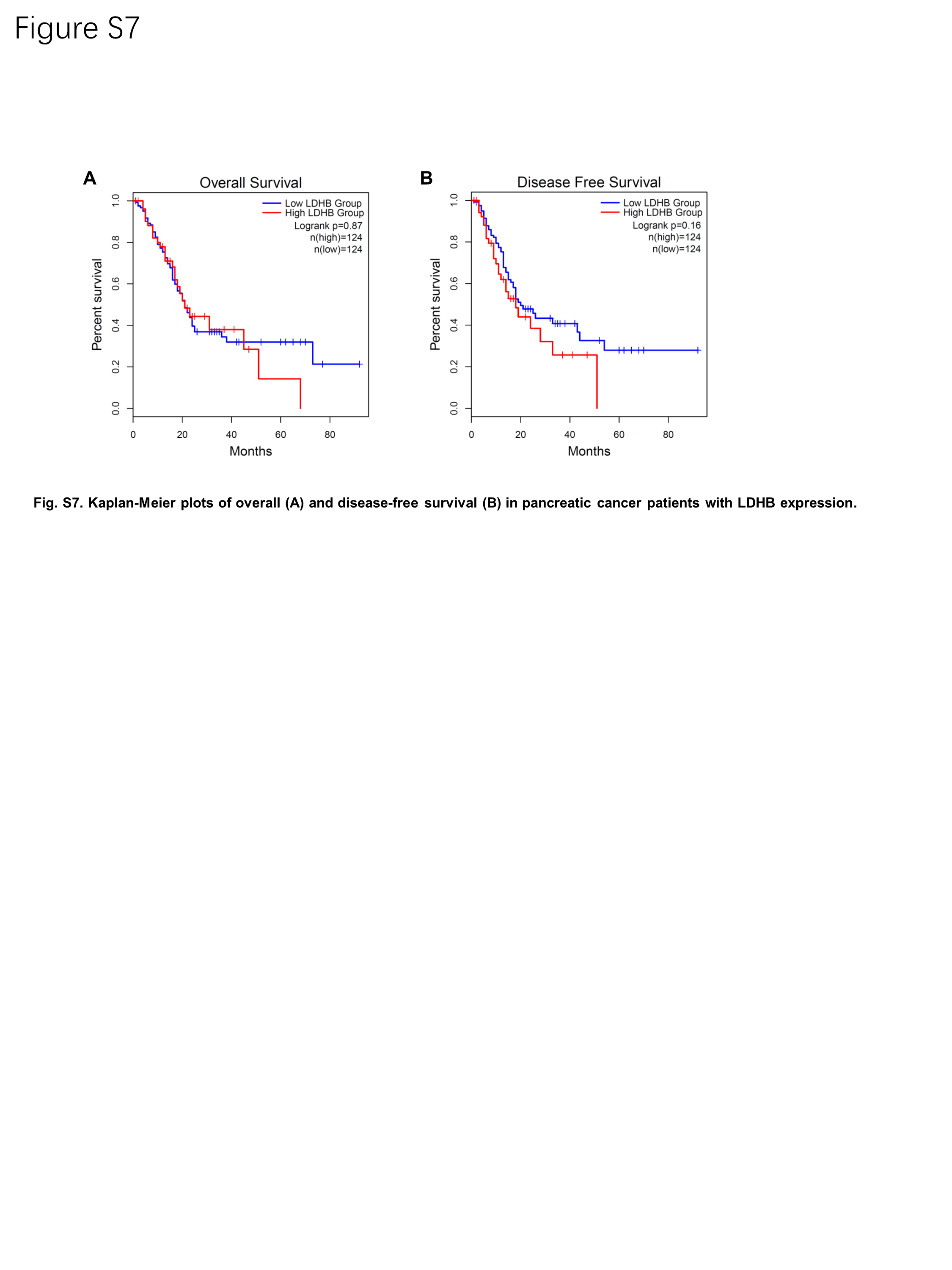

Supplement: Supplementary file 7 [file Image_7.tif]
